# 

**Published:** 1912-05

**Authors:** 


### CONTENTS.

ORIGINAL COMMUNICATIONS:—

Extra-Uterine Pregnancy. By Floyd W. McRae, M. D., Atlanta, Ga. .......... 53

Examination of a Nervous Patient. By Hansell Crenshaw, M. D., Atlanta, Ga. .. 65

Etiology, Pathology and Treatment of Pellagra. By Geo. C. Mizell, M. D., Atlanta, Ga. .......... 67

Some Thoughts on Oral Surgery of Interest to the Physician. By Robin Adair, M. D. D.D. S., Atlanta, Ga. .......... 82

EDITORIALS .......... 86

NEWS AND NOTES .......... 89

BOOK REVIEWS .......... 107

